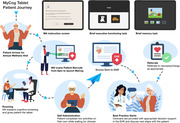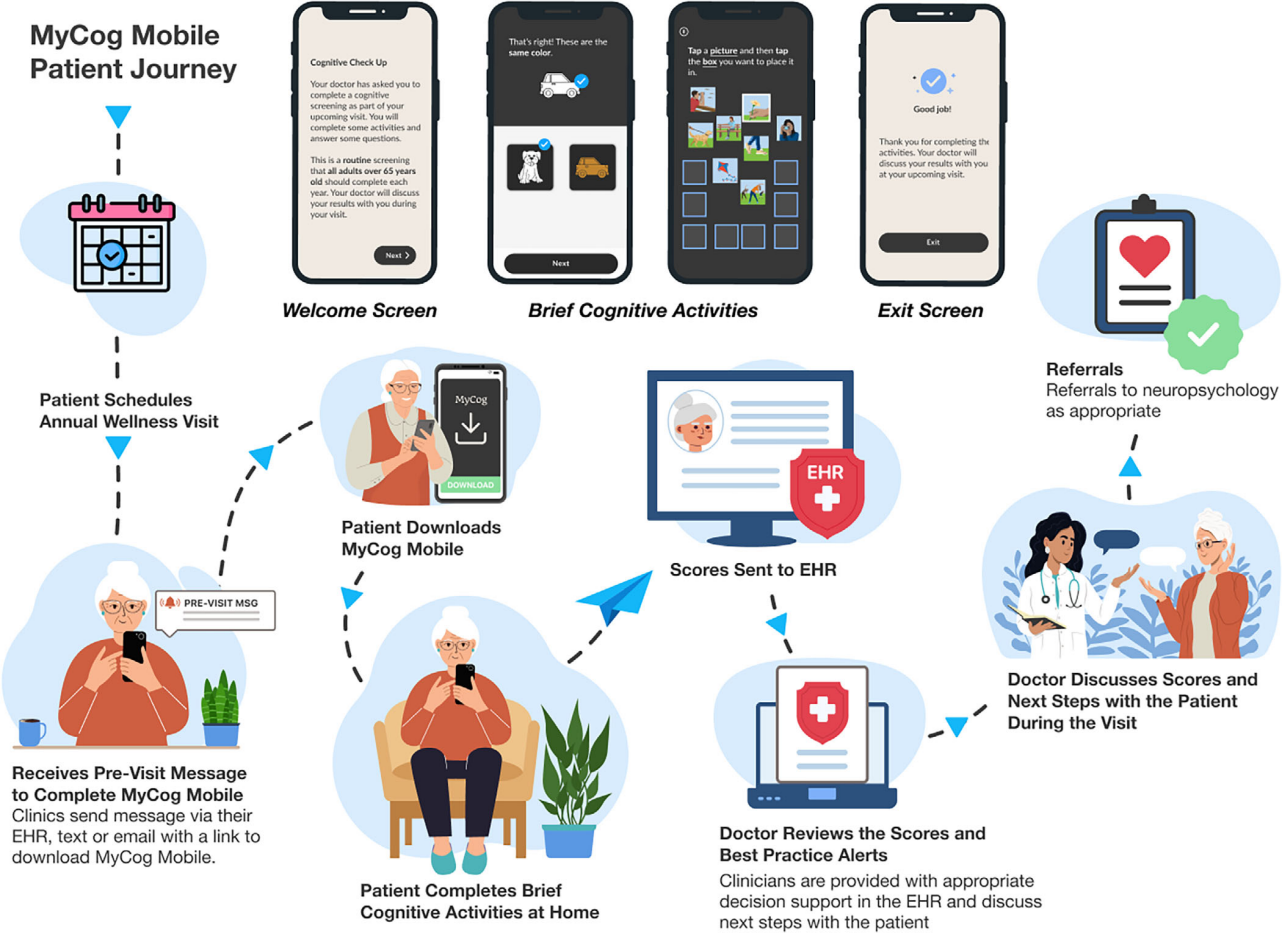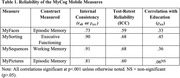# Digital Cognitive Screening in Primary Care: Feasibility, Acceptability, and Usability of the MyCog Measurement System

**DOI:** 10.1002/alz.089408

**Published:** 2025-01-09

**Authors:** Stephanie Ruth Young, Elizabeth Dworak, Greg Joseph Byrne, Julia Yoshino‐Benavente, Callie Madison Jones, Laura M Curtis, Michael S Wolf, Cindy J. Nowinski

**Affiliations:** ^1^ Northwestern University Feinberg School of Medicine, Chicago, IL USA; ^2^ Northwestern, Chicago, IL USA

## Abstract

**Background:**

Annual cognitive screening in older adults is essential for early detection of cognitive impairment, yet less than half of cases are detected in primary care. We introduce two innovative app‐based screeners that help overcome barriers to routine cognitive screening. MyCog is a tablet app that is self‐administered in person during the rooming process for a primary care visit (Figure 1). MyCog Mobile is a smartphone app that is self‐administered remotely prior to a primary care visit (Figure 2), which can save clinicians even more time during an in‐person visit. We present evidence from two studies: 1) an evaluation of the adoption and usability of MyCog in primary care; and 2) a pilot study of the reliability and usability of MyCog Mobile.

**Method:**

In Study 1, the implementation of MyCog in 10 primary care clinics was assessed as part of a pragmatic trial evaluating cognitive screening for adults aged 65+ during Annual Wellness Visits (AWVs). Using EHR data, we assessed adoption via the portion of patients who completed MyCog. In Study 2, 51 adults 65+ self‐administered MyCog Mobile remotely on their own smartphones twice about two weeks apart and rated its usability on the Simplified System Usability Scale (S‐SUS). We examined the reliability, correlations with education, and usability ratings.

**Result:**

In Study 1, of the 3921 eligible patients, 1263 completed MyCog (32.2%), with individual clinic compliance ranging from 11% to 68% of patients. Feedback revealed several addressable clinician and patient barriers, including patient frustration on memory tests, unclear test progress, and need for a Spanish version, and minor design flaws. In Study 2, all participants successfully completed each measure, and reliability metrics were broadly acceptable (Table 1). The MyPictures and education correlation was lower than expected. Ratings on the S‐SUS indicated good usability of MyCog Mobile (M = 73.78, SD = 20.11).

**Conclusion:**

MyCog demonstrated good adoption by primary care clinics in a pragmatic trial, which may be improved through design and implementation changes. Some limitations of MyCog may be addressed with MyCog Mobile, which demonstrated preliminary feasibility evidence. Practice implications and next steps are discussed.